# Cure for the itch: current clinical standards and therapies in allergic eczema

**DOI:** 10.3389/falgy.2025.1569292

**Published:** 2025-04-03

**Authors:** Jennifer E. Lazor, Bree A. Bozsoki, Pranay Bharadwaj

**Affiliations:** Biotherapeutics Discovery, Boehringer Ingelheim Pharmaceuticals Inc., Ridgefield, CT, United States

**Keywords:** allergic eczema, biomarkers, clinical research, skin disorders, allergy, IgE-mediated disease, atopic dermatatis, phototherapy

## Abstract

Allergic Eczema (AE) is a chronic, relapsing skin condition that significantly affects the quality of life of the AE patients and their caretakers. Decades of scientific and clinical research has helped understand the highly complex underpinnings of AE presentation wherein a multitude of variables, including the conspicuous variables such as environmental allergens, immunological triggers, genetic predisposition of individuals, to more nuanced socio-economic status, play an important part. Given the complexity of the disease, it is imperative to develop biomarkers enabling early and reliable clinical identifications and help in the active management of the disease, thereby minimizing the impact and burden of the disease on the patients. In this mini review, we provide a brief overview of AE, affected demographics, variables that trigger its onset, and summarize the discovery of various clinical biomarkers such as total and specific serum IgE levels, Th2 cytokine levels, filaggrin (FLG) mutations, periostin levels in skin, etc. that have been developed over the years to further improve the state of clinical monitoring of AE presentation and progression. Lastly, we also provide an overview of the clinical interventions and therapies, such as topical agents, phototherapy, and biologics, that are available to the patients to manage AE-related complications. While we have vastly improved the standard of care and diagnosis for the AE patients, there are still many unmet needs such as developing non-invasive, effective, and reliable clinical predictors and biomarkers which can usher better personalized treatments and provide a better quality of life to affected demographics.

## Introduction

Eczema is a cluster of skin conditions often characterized by inflammation, dry skin, rashes, scaly patches, and itchiness (1). Eczema affects people of all skin tones and types. In Individuals with a lighter skin tone, manifestation typically occurs in the form of erythema and skin inflammation. Eczema patients who are people of color experience brown, purple, ashen, or a gray skin tone. Though eczema affects people of all races and ethnicities, some groups are more likely to be affected (2, 3).

Around 31.6 million people, or 10% of the total population of the United States of America, suffer from some type or form of eczema. Within this population, the Asian and Pacific Islanders and Native Americans are the most affected demographics and tend to have the highest likelihood to develop eczema at 13% incidence rate, closely followed by the white population at 11%, and the black population with 10% (4).

Allergic eczema (AE) refers to a specific form of eczema which is often associated with pruritis and other IgE associated disorders such as food allergies, asthma, etc. The highest prevalence of eczema is during early childhood, with a typical onset prior to 5 years old which resolves by adulthood; however, it can re-appear later in life or continue throughout adulthood (5). Besides biological age, the gender of the person also has a bearing on the eczema incidence rates. Women have a higher incidence of eczema compared to men (8.9% and 5.7, respectively) (6). The gender discrepancy in eczema manifestation that leads to a higher incidence of allergic eczema (AE) in women is often attributed to menopause related changes. During menopause, the estrogen levels decline leading to changes in the skin which make it more prone to eczema manifestation (7).

## Causes of allergic eczema

AE pathophysiology is highly complex but is thought to primarily develop from a combination of genetic (8), immunological (9), and environmental (10) variables. Food hypersensitivity may also cause or exacerbate atopic dermatitis in 10% to 30% of patients; the majority being caused by sensitivities to egg, milk, peanuts, soy, and wheat (11, 12).

AE is one of the most common chronic, inflammatory diseases afflicting 11.3%–12.7% of children and 6.9%–7.6% adults in the USA (13)—with *pruritus* or skin barrier defects observed as the most common symptom in the patients (14). In AE, it is common to observe an immunological response imbalance, which usually results in an epidermal barrier defect, IgE mediated hypersensitivity, and other related conditions (15, 16).

Over the years, numerous scientific studies have explored the interplay of genetics and immune responses in driving AE development and flare-ups. Some of these studies looked at (i) atopic march—the early life presentation of the atopic disease and its progression with time (17), (ii) studies linking increased levels of total serum IgE and IgEs specific for environmental and food allergens (18), (iii) studies linking the incidence of AE with the loss of function mutations in the gene coding filaggrin (FLG) (19)—a filament-aggregating protein in skin that is responsible for binding the keratin fibers and maintaining the integrity of the skin and its barrier function. A common highlight shared by these studies was the role of skin as a peripheral lymphoid organ (20), and the importance of skin integrity in determining the severity of AE onset and progression (21, 22).

These immunologic and genetic studies have led to better patient stratification via the identification of other related traits or sub-phenotypes of AE (23, 24) allowing for better standard of care and clinical identifications. While the complete understanding of the pathophysiology of AE still eludes us, the high degree of coincidence of skin barrier dysfunction and immune dysregulation observed in clinical studies suggest their potential role in the etiology of disease progression in AE patients.

Reviews by Umehara et al. (25) and Sroka-Tomaszewska et al. (9) provide a comprehensive background on the complexities of AE, its possible causes and immunological underpinnings behind some of the commonly observed symptoms.

## Factors affecting the severity of allergic eczema

**Figure 1:**
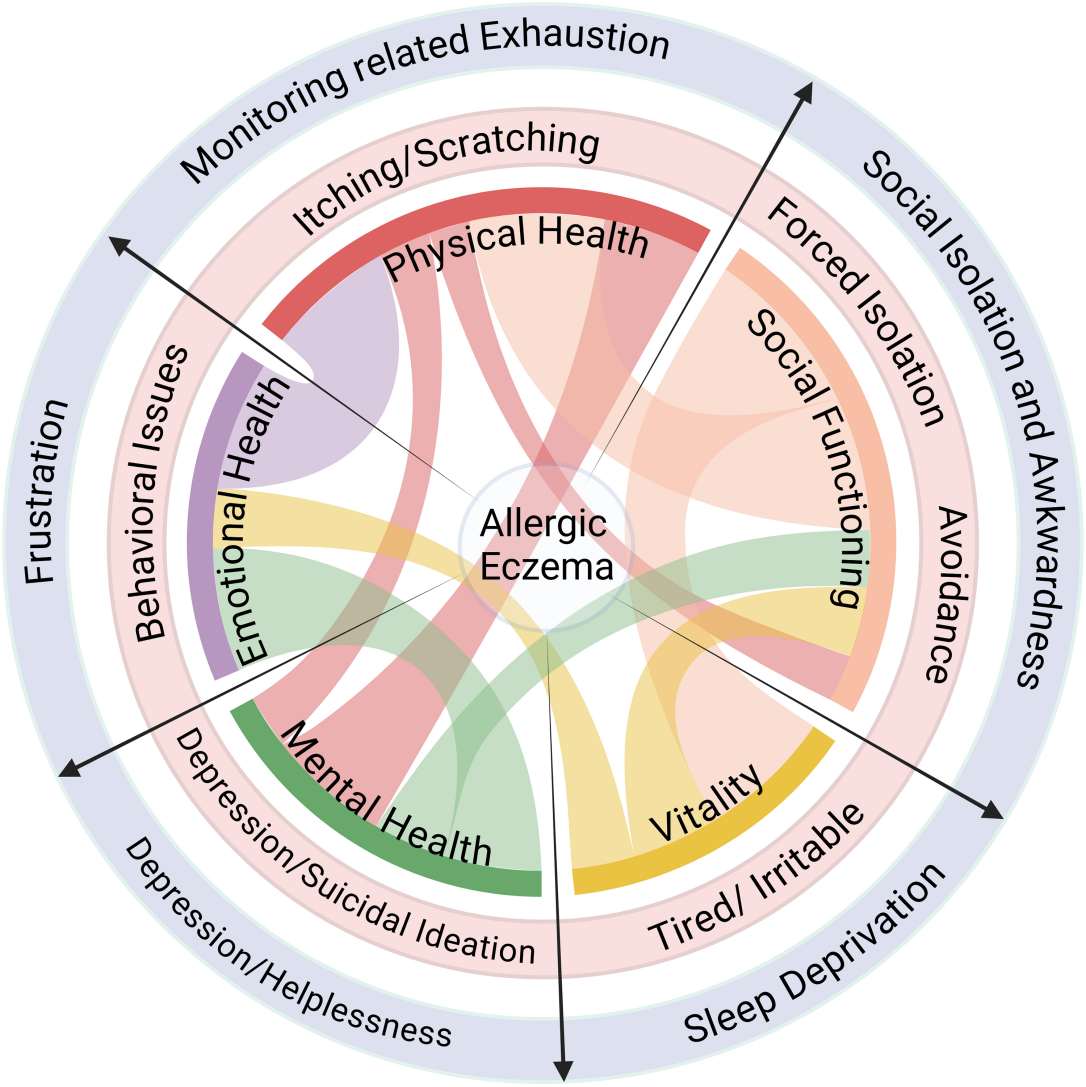
Impact of AE on quality of life. AE affects multiple facets of life and compromises the holistic well-being of the patient (light red circle) as well as their caretakers (light purple circle). Created with Biorender.com.

AE significantly affects the quality of life of both the patient and their caretakers making disease management difficult without proper planning and support. An overview of the impact and multi-faceted effects of AE disease, on patients and their caretakers alike, is provided in Figure 1.

Insomnia is frequently associated with AE (26, 27), which often leads to increased itching urges at night further disrupting sleep. Melatonin production is reduced by the lack of sleep which inhibits the body's ability to decrease temperature, resulting in the increased skin temperature during sleep, causing itching episodes (28). Irregular sleep habits can cause systemic imbalances, exhaustion, and impact the overall quality of life of the patients and their caretakers (27).

## Clinical presentation and diagnosis of allergic eczema

AE clinical characteristics change depending on age and disease duration. The characteristics in childhood centers on eczematous changes that are accompanied by serous papules which often cause a strong urge to itch, and upon scratching, may lead to excoriation and development of new papules (29).

When AE progresses from childhood to adulthood, the clinical presentation changes and the Skin lesions transition to a more varied phenotype compared to those in childhood, a condition referred to as Besnier's prurigo or disseminated neurodermatitis (30). Mental nervousness, continuous itch prior to abnormal skin, distribution of skin lesions, dryness of the skin, visible papillae of hypertrophy of skin pigment, and an apparent circumscribed plaque that appears in the same site as the original itch are all characteristics of a chronic condition of disseminated neurodermatitis (1, 29–33).

The predominant criteria used for AE diagnosis in clinical setting remains the Hanifin-Rajka criteria (23)—first introduced in 1980, and included almost 30 signs, symptoms, and laboratory abnormalities (34), of which 3 out of 4 major criteria and 3 out of 23 minor criteria need to be met for a conclusive diagnosis. Given challenges around the difficult interpretation of the Hanifin-Rajka criteria outside a clinical setting, the United Kingdom (UK) Working Party Criteria was introduced in 1994 (35, 36). This simplified and condensed the Hanifin-Rajka criteria for a broader application to a range of ethnic groups, with pruritus being elevated to be the sole mandatory criteria in addition to coincidence of 3 or more of other 5 major criteria. A further distillation of diagnosis criteria was observed in the millennium criteria, introduced in 1998 by Bos et al. (37), and was the first to include the presence of allergen-specific IgE as a diagnostic biomarker.

Later, in 2011 Schram et al. elaborated on the millennium criteria and proposed meeting ≥5 of the criteria outlined in the original millennium criteria (35). A head-to-head assessment of the modified millennium criteria with the established UK working party criteria and Hanifin-Rajka criteria, over a cohort of 201 patients and observed that the modified minimum criteria showed a sensitivity of 81.8% with a specificity of 98.8%, compared to 100% and 48.8% for the Hanifin-Rajka criteria and 97.7% and 72.9% for the UK working party criteria (35, 38). In 2014, the American Academy of Dermatology laid out the guidelines for AE diagnosis and assessments which emphasized the role of hallmark features of AE opposed to features that are non-specific while excluding the conditions that mimic AE, such as scabies, seborrheic dermatitis, psoriasis, etc. (23).

To determine the severity of the disease in the clinic, the following two tests are commonly used and have been validated: Scoring Atopic Dermatitis (SCORAD) index, and the Eczema Area severity index (EASI) (39, 40). These tests are typically used to measure the extent of erythema, edema, papulation, excoriation, and the degree of lichenification. Additionally, other factors, such as pruritus and loss of sleep, are incorporated into the diagnosis and severity assessment (22, 26, 27).

Many of the current clinical practices for diagnosing cases of AE rely on previous clinical learnings and established biomarker tracking in order properly identify and stratify the patient and recommend therapeutic interventions accordingly.

## Biomarkers of allergic eczema

Identification and characterization of appropriate biomarkers allows for a precise understanding of multi-factorial disease such as AE and its complex underpinnings. This understanding is crucial for enabling tailored therapeutic approaches for individual patient profiles. The utility of such biomarkers ranges from the assessment of disease severity to predicting flares and guiding treatment decisions, which in turn aids in improvement of patient outcomes overall.

Monitoring critical biomarkers can also help in tracking the therapeutic responses of the treatment and allows for a more involved and dynamic approach in clinics, where timely modifications in treatment plan can ensure rapid alleviation of the disease condition.

The last few decades of intense research around AE and its underpinnings have allowed clinicians to come up with several biomarkers that are used in clinics to track the disease state. Some of these commonly used biomarkers have been summarized in Table 1.

**Table 1:** Various biomarkers currently used in AE diagnosis and tracking.

Immunological biomarkers	
Total and specific serum IgE levels	Numerous studies have established that patients with allergic eczema frequently exhibit elevated total serum IgE levels (32, 33). Leung et al. demonstrated that high IgE levels correlate with increased disease severity and are indicative of atopic sensitization to environmental allergens (33). Specific IgE responses to common allergens [e.g., dust mites (76), pollen (77)] have also been linked to the exacerbation of AE symptoms.
Eosinophil cationic protein (ECP)	ECP is a marker of eosinophilic inflammation associated with allergic diseases (78). Early studies, such as those by Hozumi et al. (79), reported elevated ECP levels in AE patients, suggesting its utility as an inflammatory marker reflective of disease activity.
Genetic Markers	
Filaggrin Mutations	Filaggrin (FLG) is crucial for maintaining skin barrier integrity. Research by Brown et al. Identified mutations in the FLG gene as a significant risk factor for developing allergic eczema, particularly in patients of European descent (80). This study linked genetic predisposition to the clinical presentation of atopic dermatitis and highlighted the importance of barrier dysfunction in AE pathogenesis.
Cutaneous biomarkers	
Skin barrier function	Studies assessing skin hydration and trans-epidermal water loss (TEWL) have consistently shown that impaired skin barrier function is a hallmark of atopic eczema like conditions (21, 81). Research conducted by Flohr et al. Explored the relationship between TEWL and disease severity, demonstrating that increased TEWL correlates with worse clinical outcomes (15, 82).
Periostin	Periostin has been implicated in the regulation of inflammation in AE (15, 32, 41, 83). A study by Kou et al. found that periostin levels in the skin are positively associated with clinical severity, suggesting it may serve as a potential biomarker for chronic inflammation in AE (84).
Cytokine profiles	
Th2 cytokine dominance	A pivotal characteristic of allergic eczema is the Th2-skewed immune response (14, 29, 85–87). Research has documented elevated levels of cytokines such as IL-4, IL-13, IL-17 and IL-5 in AE patients (15, 22, 32, 70, 73, 88–90).

## Limitations of historical research

In the past, significant progress was made in identifying and understanding various biomarkers associated with allergic eczema. Key studies highlighted the roles of immunological factors, genetic predispositions, cutaneous protein levels, and cytokine profiles in the disease's pathogenesis and management (21, 24, 32, 41). However, further validation and exploration of these biomarkers are needed to enhance their clinical applicability and improve patient outcomes.

Despite the identification of several potential biomarkers, significant limitations persist including issues with specificity, sensitivity, and inconsistency of findings across diverse populations. Many biomarkers were not widely validated for routine clinical use, emphasizing the need for further research into more reliable and clinically applicable biomarker candidates.

Research in the past decade has significantly advanced the understanding of biomarkers in AE. Key findings include the identification of genetic (e.g., FLG mutations), immune (e.g., Th2 cytokines), and skin barrier-related biomarkers (e.g., filaggrin, periostin) that are pivotal for diagnosing, monitoring, and personalizing treatment for AE. Additionally, the role of the microbiome, serum biomarkers, and exosomes is gaining attention as potential tools for precision medicine in AE. This ongoing research is improving both the management of the disease and the development of targeted therapies, ultimately aiming for more effective and individualized treatment strategies.

## Treatment modalities

### Traditional

Traditional therapy for AE could be split into two types of treatments: topical or systemic. Topical agents include moisturizers, corticosteroids, antimicrobials, wet wrap therapy, and calcineurin inhibitors (1, 23, 33, 42, 43). Systemic treatments are used for more severe forms of AE. These treatments consist of systemic corticosteroids, cyclosporine, azathioprine, phototherapy, and more (21, 42, 44–46).

#### Topical therapies for AE

##### Topical agents

Use of topical emollients (lotions, creams, hydrating gels, sprays, and ointments) and anti-inflammatory agents (antihistamines, phosphodiesterase inhibitors, calcineurin inhibitors) can achieve short term control of acute symptoms and clinical improvement in mild allergic eczema and dermatitis skin diseases. Topical agents are more effective in controlling mild disease conditions as opposed to moderate-to-severe stage of AE (42). Topical corticosteroids (TCS) suppress the release of proinflammatory cytokines and inhibit antigen processing (47, 48). TCS is efficient as a maintenance therapy to help reduce the number of relapses. TCS is often clinically used along with moisturizers as the topical agent in wet wrap therapy (WWT) which is an efficient method to reduce and manage severe dermal flares.

Both traditional and prescription moisturizers have shown to decrease the symptoms of AE. They soften the skin and reduce the evaporation of water which prevents skin from drying out. These therapeutic interventions are the primary treatment for mild forms of AE but are generally included in the regimen for moderate-to-severe forms, due to the reduction of inflammation and alleviation of physical discomfort.

#### Systemic therapies for AE

##### Systemic corticosteroids

Systemic corticosteroids are generally reserved as a short-term bridging therapy for acute and severe flare-ups, as they can have both short- and long-term adverse effects. A systematic corticosteroid treatment consists of an oral corticosteroid with or without an additional immune suppressant such as cyclosporine, methotrexate, azathioprine, and mycophenolate mofetil (44). Three distinct clinical trials performed over varied ages and gender concluded that short-courses of systemic corticosteroid interventions could provide relief to patients with severe flare-ups or while awaiting response to other therapeutic interventions (49–51).

##### Phototherapy

Phototherapy as a therapeutic intervention was first introduced in 1925 in the Mayo Clinic by William Goeckerman for treatment of psoriasis (52, 53) and falls under the umbrella of systemic therapeutic options available for patients. Phototherapy treatment decreases cutaneous inflammation and is beneficial for moderate to severe AE, with limited systemic side effects (54, 55) compared to the alternatives and is suitable for patients of all age demographics.

Phototherapy, while very effective for some patients, has its limitations such as inconvenience and potential adverse effects. An inconvenience includes the access to in-office treatments, a 3-times per week regimen for patients, which can be difficult to maintain. Rare potential adverse effects include increased risk of skin cancer and flare-ups from the excessive heat (45, 56, 57). Some studies have shown that narrowband UVB can be considered an efficient treatment with patients suffering from chronic AE exhibiting at least a 50% reduction in SCORAD index scores with the phototherapy. The disease activity reversal was associated with the elimination of the inflammatory leukocytes (58).

### Novel therapeutics modalities

Learnings from the clinical studies and research have driven the introduction of novel drug delivery systems and therapeutics that address the AE-related symptoms, which were previously without a cure or as effective. While these therapies do provide a promising outlook for the patients of AE, the data on their efficacy and applicability for various demographics is limited for some. A detailed review by Waligóra-Dziwak et al. provides an excellent primer on various novel biologics currently in Phase III and Phase IV clinical trials (59).

#### Vehicles for drug delivery

Currently, topical application of creams or ointments are the predominant vehicle for delivery of drugs for AE. Novel delivery approaches such as electrospun patches (60, 61), sprays (62), liposomes (63), nanoparticles (64), and lasers have recently been developed to enhance transdermal delivery with a focus on increasing treatment adherence while minimizing the side effects.

#### Biological modalities

##### Small-molecule inhibitors

New biologics and small-molecule inhibitors are being developed against key molecules for addressing the full spectrum of AE disease manifestations. Novel topical drugs that are currently approved or in late-stage clinical trials include, but are not limited to, Janus family protein kinases (JAK) inhibitor (ruxolitinib) (65), phosphodiesterase-4 inhibitors (crisaborole) (66, 67), and roflumilast (68).

##### Monoclonal antibody based therapeutics (mAb Tx)

Monoclonal antibodies that target specifically offer a focused and highly efficacious therapeutic option. mAb Tx against cytokine receptor IL-4Rα (e.g dupilumab, CM310, and CBP201) have also been developed to address unmet AE-related conditions. Th2 cytokines, IL-4 and IL-13, have a significant impact on the role in pathogenesis of allergic eczema (69–71). The IL-4Rα binds the IL-4 and IL-13 cytokines via the formation of type I and type II receptors, respectively. This binding event leads to the activation of the JAK inhibitors and its downstream counterpart—signal transducer and activator of transcription (STAT)—that acts as an activator of transcription leading to downstream signaling pathways associated with IL-14 and IL-13 (72).

JAK inhibitors can block multiple cytokine-signaling pathways and is thus the preferred target when aiming for broad immunomodulation (73). Historically, topical JAK inhibitors have exhibited fewer adverse events compared to oral JAK inhibitors (69); however, the US Food and Drug Administration (FDA) recently placed a black box warning on this class of medications due to safety concerns based on data from studies investigating tofacitinib in patients with rheumatoid arthritis (74). This news along with the inadequate guidance on communicating the merits and drawbacks of JAK inhibitors has been a source of hesitation for the dermatologists in recommending this treatment modality (75).

## Conclusion

Eczema cases have seen a steady rise in the past decades with about 10% of the total population of the United States exhibiting some form of eczema. Incidences of AE have been closely linked to genetic and environmental factors leading to a loss of proper regulation of the housekeeping immunological functions, which results in eventual compromise in the dermal integrity and presentation of AE symptoms. Most of the AE patients experience a chronic, relapsing disease course with hallmark remissions and flare-ups. Clinical presentation of AE can be varied and nuanced, and early and accurate clinical diagnosis is imperative in ensuring an appropriate standard of care is provided to the patient. To this end, several biomarkers have historically been identified which have had a wide range of successes and clinical applications. While we have come a long way in terms of the biomarker reliability and effectiveness, there is still a huge scope of improvement in their performance and applicability across the multiple facets of AE. Recent advancements in therapeutic and palliative interventions have allowed patients to manage flare-ups and minimize the impact of AE on their quality of life. These interventions range from (i) topical emollients to moisturize and soothe the affected skin areas, (ii) phototherapy to decrease cutaneous inflammation with very limited side effects, and (iii) biologic modalities, such as JAK inhibitors and antibody-based therapeutics, which directly address the cytokine signaling pathways thereby putting the brakes on the progressive inflammatory immune responses.

Developing a better understanding of the underpinnings of AE pathogenesis will allow for the development of novel treatment options with breakthrough potential, personalized treatment plans, and recovery strategies, all of which will help address the unmet needs of a huge patient demographic and allow them and their caretakers to live a more enjoyable and holistic life.
